# Homogeneous and heterogeneous photoredox-catalyzed hydroxymethylation of ketones and keto esters: catalyst screening, chemoselectivity and dilution effects

**DOI:** 10.3762/bjoc.10.114

**Published:** 2014-05-19

**Authors:** Axel G Griesbeck, Melissa Reckenthäler

**Affiliations:** 1 University of Cologne, Department of Chemistry, Organic Chemistry, Greinstr. 4, D-50939 Köln, Germany; Fax: +49(221)470 5057

**Keywords:** alkylation, carbonyl, photocatalysis, photoredox catalysis, redox, semiconductor

## Abstract

The homogeneous titanium- and dye-catalyzed as well as the heterogeneous semiconductor particle-catalyzed photohydroxymethylation of ketones by methanol were investigated in order to evaluate the most active photocatalyst system. Dialkoxytitanium dichlorides are the most efficient species for chemoselective hydroxymethylation of acetophenone as well as other aromatic and aliphatic ketones. Pinacol coupling is the dominant process for semiconductor catalysis and ketone reduction dominates the Ti(OiPr)_4_/methanol or isopropanol systems. Application of dilution effects on the TiO_2_ catalysis leads to an increase in hydroxymethylation at the expense of the pinacol coupling.

## Introduction

**Scheme 1:**
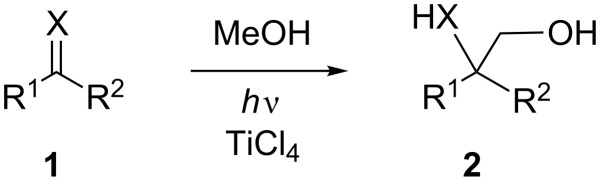
Photohydroxymethylation of carbonyl compounds and imines.

Stimulated by the principles of sustainable chemical synthesis and the progress in our understanding of catalytic and photoinduced electron-transfer processes, in recent years photoredox catalysis emerged as a new and powerful area for advanced synthesis [1–10]. There are numerous features that characterize an effective photoredox catalytic cycle: light absorption, charge separation, charge transport and annihilation as well as the use of appropriate sacrificial compounds such as electron and hole donors, elements that also appear in the natural photosynthesis, the role model for all applications. Catalysts that can function as light-absorbing and as redox-activating species must combine several features: redox-inactivity in the electronic ground states, optimal absorption properties in the near UV or visible region and appropriate redox activity in the excited states. Many potent photoredox catalysts with sufficient long-term stability are transition metal complexes with excited MLCT states that can be generated in the visible. Another important group of photocatalytic active compounds are semiconductor particles that absorb in the UV-A and near visible reagion. The widely used TiO_2_ has found numerous applications in photochemical water detoxification or surface purification because of its favourable excited-state redox properties [11]. In synthetic applications of semiconductor photocatalysis two clearly distinguishable reaction protocols were designated as type A and B by Kisch and co-workers [12–15]. In the type A process, two different products are formed from the initially formed electron–hole pair, one from the substrate radical cation that is formed from electron transfer to the semiconductor valence band hole, the other from the substrate radical anion that is formed from reduction by the semiconductor conduction band electron. Mostly, one of these steps consumes a sacrificial electron/hole donor. In type B photocatalysis, combination of the radical ions leads to a new product without the need of sacrificial components. The latter process proceeds with a high degree of atom economy [16]. We have recently demonstrated this for the azido-hydroperoxidation of alkenes, a convenient method for the synthesis of 1,2-amino alcohols [17–18]. In the field of C–C coupling reactions, the direct hydroxyalkylation of carbonyl compounds and carbonyl analogs is a demanding task because the α-CH activation of alcohols must occur in the presence of the acidic and nucleophilic hydroxy group. Thus, protection and deactivation of this group is necessary for thermal processes. In contrast to that, photochemical redox activation is possible in the presence of titanium(IV) catalysts [19–22]. As shown in a series of papers by Sato and coworkers, carbonyl compounds **1** as well as imines couple with methanol to give the 1,2-diols or 1,2-amino alcohols, respectively, when irradiated in the presence of stoichiometric or sub-stoichiometric amounts of titanium tetrachloride (Scheme 1). In order to run these reactions to completion, not less than 0.5 equivalents of TiCl_4_ were necessary which accounts for severe catalyst consumption. Furthermore, the addition of TiCl_4_ to methanol solutions is cumbersome and it is unclear what species is catalytically active. These processes have thermal counterparts in reduced titanium-mediated chemistry, e.g., the Ti(III)/*t*-BuOOH-mediated hydroxymethylation of imines [23–24].

In order to evaluate the nature of the active catalytic species in the photochemical homogenous titanium-catalyzed hydroxymethylation and to develop a truly catalytic process, we used a model reaction for catalyst screening (acetophenone/methanol) and applied the optimal homogenous reaction conditions involving titanium catalysis to other ketones and keto esters.

## Results

### Nature of the homogeneous catalytic titanium species

**Scheme 2:**
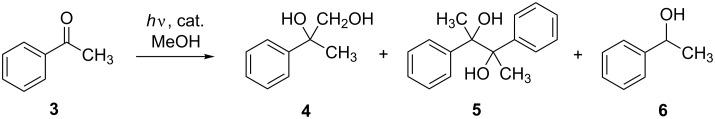
Model process: photocatalyzed acetophenone/methanol reaction.

The original protocol for photocatalytic hydroxymethylation involves titanium tetrachloride in methanol as the reactive catalyst/donor mixture and carbonyl compounds as the acceptor components. During the exothermic dissolution process of TiCl_4_ in methanol with formation of gaseous HCl, a slightly yellowish solution is formed that, after irradiation with UV-A light, turns into a bluish solution indicating the formation of reduced titanium species. Obviously, ligand exchange reactions lead to a series of chloro- and methoxy-titanium complexes that have different catalytic activities. In order to simulate the different complex stages, we applied different monomeric titanium complexes of the type TiCl*_n_*(OiPr)_4−_*_n_* (*n* = 0, 1, 2, 3) [25–27] in the model process, the irradiation of a solution of acetophenone (**3**) in methanol (Scheme 2). In the absence of any titanium species, photolysis at 254 and 300 nm, respectively, led only to trace amounts of the hydroxymethylation product **4** via a (triplet carbonyl) excited-state hydrogen-transfer process, obviously a sluggish process under these conditions (Table 1). In the presence of titanium complexes TiCl*_n_*(OiPr)_4−_*_n_*, coupling and reduction products **4** and **6** were formed without pinacol **5** formation (Scheme 2).

**Table 1:** Homogeneous sensitizer variation for the acetophenone model reaction.

catalyst^a^	irrad. wave-length (nm)	yield **4** (%)^b^	yield **5** (%)^b^	yield **6** (%)^b^

none	300 254	<5^c^ <5^c^	– –	– –
TiCl_4_	300 254	33 34	– –	– –
TiCl_3_OiPr	300 254	31 47	– –	– –
TiCl_2_(OiPr)_2_	300 254	0 60	– –	– –
TiCl(OiPr)_3_	300 254	<5^d^ <5^d^	– –	– –
Ti(OiPr)_4_	300 254	– –	– –	– 46
Ti(OiPr)_4_/iPrOH^e^ Ti(OiPr)_4_/BF_3_^d^ Ti(OiPr)_4_/AlCl_3_^d^	254 254 254	– 45 49	– – –	43 – –

^a^0.375 mmol catalyst in methanol (6 mL), 0.75 mmol acetophenone, irradiation time 72 h, rt; ^b^isolated yields ; ^c^trace amounts detected in ^1^H NMR; ^d^0.75 mmol added in methanol; ^e^0.375 mmol cat. in isopropanol.

**Scheme 3:**
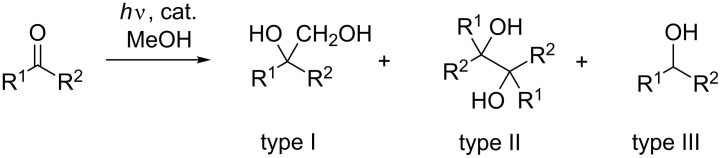
Photocatalyzed acetophenone/methanol reaction: types I–III.

In the presence of the tetraalkoxide Ti(OiPr)_4_, only the reduction product **6** was detected which demonstrates that chlorotitanium complexes are crucial for the desired reaction path. The results from TiCl_4_ and TiCl_3_OiPr were nearly identical at both wavelengths whereas for TiCl_2_(OiPr)_2_ catalytic activity was preserved only for the 254 nm irradiation. These results show that different catalytically active species must exist that can be excited in different wavelength regions. TiCl(OiPr)_3_ showed no activity at all, meaning that no further ligand exchange to a tetraalkoxide did occur from this complex. The optimal results concerning reaction time and yields were observed for the TiCl_2_(OiPr)_2_ species. The apparently different catalytic activity of titanium tetraalkoxide complexes could be switched back to hydroxymethylation in the presence of additional strong Lewis acids such as AlCl_3_ or BF_3_. These compounds alone did not show catalytic activity in methanol, only in combination with Ti(OiPr)_4_. With the optimal homogenous reaction conditions in hand, we applied several other aromatic and aliphatic open chain and cyclic ketones as substrates (Scheme 3). Even benzophenone, a notorious pinacol forming substrate, gave moderate yields of the hydroxymethylation product **7**. Among the aromatic ketones, *para*-fluoroacetophenone was the most reactive ketone. Excellent yields were obtained for 2-pentanone where the product **18** was isolated without purification after extraction. For comparison, the results from the Ti(OiPr)_4_ catalysis are included in Table 2. Additonally, the comparision with the heterogeneous TiO_2_ photolyses demonstrates that under semiconductor conditions pinacolization becomes the major path, but only for aromatic ketones. Aliphatic ketones did not show conversion under TiO_2_ photolyses.

**Table 2:** Substrate variation under optimized conditions.

entry	Substrate	TiCl_2_(OiPr)_2_^a^	TiO_2_ P25^b^		Ti(OiPr)_4_^a^
		yield (%)^c^ type I	yield (%)^c^ type I	yield (%)^c^ type II	yield (%)^c^ type III

1	Ph(CO)CH_3_	60 (**4** [28])	1 (**4**)	82 (**5** [29])	46 (**6** [30])
2	Ph(CO)Ph	28 (**7** [31])	20 (**7**)	71 (**8** [24])	50 (**9** [32])
3	2’-F-Ph((CO)CH_3_	56 (**10**)	–	54 (**11**)	40 (**12** [33])
4	4’-MeO-Ph(CO)CH_3_	–	–	23 (**13** [34])	–
5	4’-NO_2_-Ph(CO)CH_3_	19 (**14** [35])	–		–
6	4’-Me-Ph(CO)CH_3_	27 (**15** [36])	6 (**15**)	54 (**16** [34])	16 (**17** [37])
7	C_3_H_7_(CO)CH_3_	92 (**18** [38])	–		–
8	cyclohexanone	38 (**19** [39])^d^	–		–

^a^0.75 mmol ketone in methanol (6 mL), 0.5 equiv cat., irradiation time 72 h, λ = 254 nm (TiCl_2_(OiPr)_2_) and 300 nm (Ti(OiPr)_4_), rt; ^b^0.75 mmol ketone in methanol (6 mL), 1.4 wt % TiO_2_ P25, irradiation time 48 h irradiation, λ = 350 nm, rt; ^c^isolated yields; ^d^mixture consisting of 9% 1,2-diol and 29% acetalization product with cyclohexanone (**20**).

**Scheme 4:**
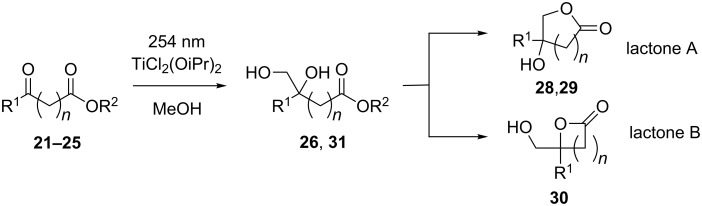
Photohydroxymethylation and subsequent lactonization of keto esters.

These conditions were applied to keto ester substrates that feature an additional trapping site for the primary hydroxy group. We envisaged the formation of lactones from the corresponding hydroxymethylation products (Scheme 4). Methyl benzoylformate (**21**) gave a mixture of pinacol and the 1,2-diol without lactone formation. The higher homologs (Table 3, entries 2–4) resulted in the corresponding lactones with ring sizes of 5 and 6, respectively, with the δ-keto ester (Table 3, entry 4) leading to the δ-pentyrolactone and not the corresponding seven-membered lactone. The primary product from hydroxymethylation was also isolated from the reaction of the ε-keto ester **25** [40].

**Table 3:** Photohydroxymethylation of keto esters: 1,2-diol and lactone formation.

entry	compound^a^	R^1^	R^2^	*n*	1,2-diol (%)^b^	lactone A (%)^b^	lactone B (%)^b^

1	**21**	Ph	Me	0	15 (**26** [41])^c^	–	–
2	**22**	Ph	Et	1	–	56 (**28** [42])	–
3	**23**	Me	Et	2	–	54 (**29**)	–
4	**24**	Me	Et	3	–	–	28 (**30** [43])
5	**25**	Me	Me	4	44 (**31**)	–	–

^a^0.75 mmol keto ester in methanol (6 mL), 0.5 equiv TiCl_2_(OiPr)_2_, irradiation time 72 h, λ = 254 nm, rt; ^b^isolated yields; ^c^additionally 11% of the corresponding pinacol **27** was formed.

### Heterogeneous and dye-sensitized photocatalysis

**Scheme 5:**
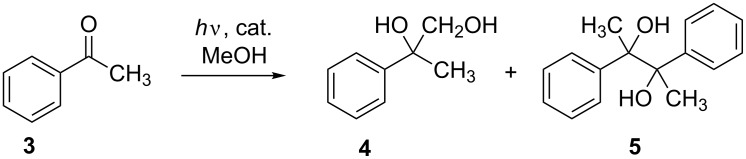
Model reaction for heterogeneous and dye-sensitized catalysis.

The results with the semiconductor particle TiO_2_ P25 under low catalyst loading conditions appear in Table 2 for a series of ketone substrates. In order to explore the catalyst profile we tested other reaction conditions for the TiO_2_ catalysis, other metal-containing heterogeneous and homogeneous catalysts as well as the classical organic PET catalyst 9,10-dicyanoanthracene (DCA). The results are summarized in Table 4 for the model reaction of acetophenone in methanol (Scheme 5). Except for the Ru(bpy)_3_Cl_2_ system, all catalysts enabled a high degree of conversion and high yields of the pinacol **5** were detected. The hydroxymethylation product **4** was detected only in few experiments with a maximum yield of 6% from one TiO_2_ experiment. The best results were obtained for TiO_2_ P25 catalysis in the presence of molecular sieves (Table 4, entry 3). In all cases, the pinacol diastereoisomers were formed in nearly equal amounts. Also the change in irradiation wavelength did not lead to substantial changes in conversion and chemoselectivity. The formation of formaldehyde as the final oxidation product was proven qualitatively (colorless precipitation of polyformaldehyde was observed in most experiments) and by a GC–MS online detection of monomeric formaldehyde.

**Table 4:** Heterogeneous sensitizer variation for model reaction in comparison with optimized homogeneous conditions for model process.

entry^a^	catalyst	loading	conversion (%)^b^	yield **4** (%)^b^	yield **5** (%)^b^

1	TiO_2_ P25	2.8 wt %	77	–	66
2		2.8 wt %^c^	76	–	58
3		2.8 wt %^d^	96	3	83
4		2.8 wt %^e^	71	–	48
5		2.2 wt %	89	6	79
6		1.4 wt %	95	1	82
7		3.3 wt %	81	2	74
8	TiO_2_-pigment	2.8 wt %	89	–	69
9	zinc white	2.8 wt %	79	–	69
10	WO_3_, <100 nm	2.8 wt %	98	–	70
11		1.4 wt %	95	1	75
12	Fe_2_O_3_, <50 nm	2.8 wt %	92	–	75
13		1.4 wt %	95	–	80
14	ZnO, 6% Al doped, <50 nm	2.8 wt %	63	–	46
15	InSnO, <50 nm	2.8 wt %	65	–	45
16	Ir(ppy)_3_	2.5 mol %	91	–	50
17		2.5 mol %^f^	65	–	55
18		0.5 mol %	89	–	82
19	Ru(bpy)_3_Cl_2_	2.5 mol %	27	–	1
20	DCA	2.5 mol %	95	1	94
21		0.5 mol %	96	1	92
22	none	–	<5%	–	–

^a^0.75 mmol acetophenone in methanol (6 mL), irradiation time 24 h, λ = 350 nm, rt; ^b^determined by GC; ^c^λ = 300 nm; ^d^600 mg molecular sieves were added; ^e^100 µL H_2_O was added; ^f^Ir(ppy)_3_ regained from entry 16.

From these results, we reasoned that methanol oxidation is the primary event (e.g., from the DCA results) and acetophenone reduction follows resulting in the corresponding hydroxybenzyl radicals that couple to give the pinacol **5**. Under this assumption, a decrease in ketone concentration should favour radical combination of the hydroxybenzyl and the (more reactive and easier oxidizable) hydroxymethyl radicals. As shown in Table 5, this is actually the case for the semiconductor particle TiO_2_ P25 catalysis. If acetophenone is added to the photolysis solution constantly over a period of 24 h, the absolute amount of hydroxymethylation product **4** can be increased to 40%.

**Table 5:** Chemoselectivity modification by application of a dilution effect.

entry	drop rate	TiO_2_ in methanol (30 mL)	reaction time	conversion (%)	yield **4** (%)	yield **5** (%)

1^a^	0.28 mmol/h	15 mg	18.7 h	92	36	52
2^a,b^	0.28 mmol/h	15 mg	18.2 h	82	25	38
3^a^	0.21 mmol/h	15 mg	24 h	95	33	55
4^a^	0.21 mmol/h	10 mg	24 h	89	13	60
5^c^	0.16 mmol/h	10 mg	24 h	96	40	50

^a^5 mmol acetophenone dissolved in methanol (10 mL) was slowly added to a TiO_2_ P25 (15 mg/ 10 mg) suspension in methanol (30 mL) irradiated (300 nm) at 15 °C; ^b^TiO_2_ P25 suspension was cooled to −5 °C; ^c^3.72 mmol acetophenone dissolved in methanol (10 mL) was slowly added to a TiO_2_ P25 (10 mg) suspension in methanol (30 mL) irradiated (300 nm) at 15 °C.

## Discussion

**Scheme 6:**
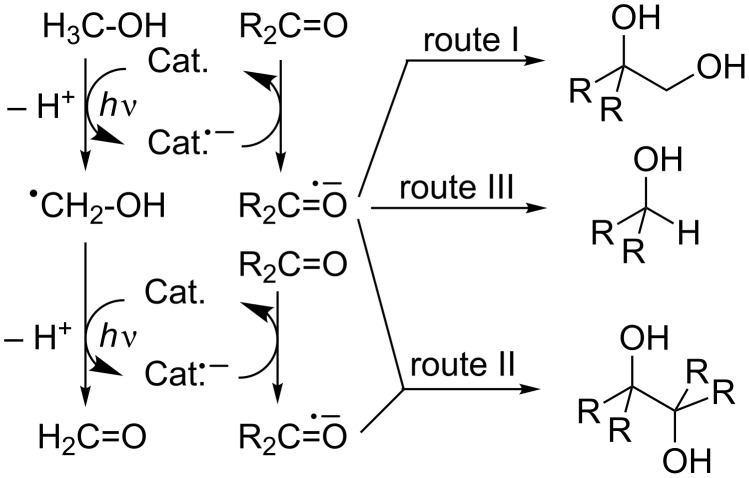
Product forming routes I to III for photoredox catalysis of methanol/carbonyl compounds.

Three product-forming routes can be assumed for the three classes of products observed in the study (Scheme 6): hydroxymethylation (route I), pinacolization (route II) and reduction/hydrogenation (route III). The crucial primary step for all processes is methanol oxidation [44]. By using appropriate reaction conditions, every route can be switched on exclusively. Hydroxymethylation is favoured if both hydroxyalkyl radicals are generated in close proximity by a coupled electron transfer/back transfer process. According to this expectation, the optimal conditions for route I are fulfilled for TiCl_2_(OR)_2_, a species that is capable of oxidizing methanol in the excited state and simultaneously acting as a ground-state Lewis acid that complexes the carbonyl compound. A much weaker Lewis acid such as Ti(OR)_4_ is capable of methanol oxidation but prefers hydrogen transfer at the first or second oxidation event. The pinacolization route II is favoured for heterogeneous and dye-catalyzed conditions. Interestingly, the combination of TiO_2_ P25 with an organic dye prefers largely the hydrogenation route III [45].

**Scheme 7:**
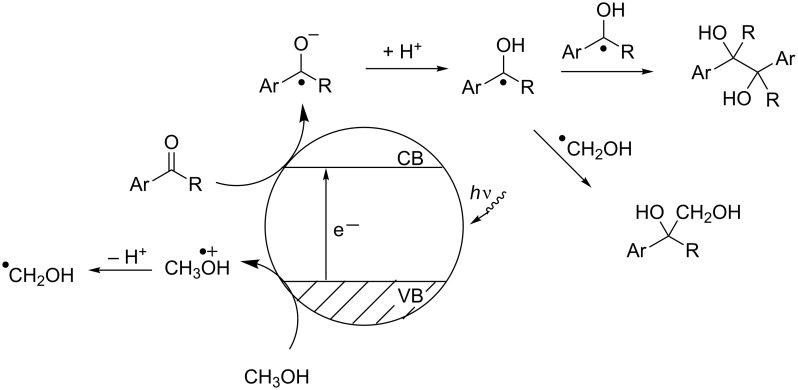
Photoredox initiated steps on semiconductor particle surfaces, CB, VB = conduction and valence band.

On the surface of the relatively large semiconductor particles, combination events are rare between the hydroxymethyl radical from methanol oxidation and the hydroxyalkyl radical from ketone reduction. Thus, further oxidation of the hydroxymethyl species to give methanol and another hydroxyalkyl radical is feasible. The combination of two hydroxyalkyl radicals is then dictated by diffusion kinetics (Scheme 7). The dilution experiments described in Table 5 indicate that the probability for pinacol formation is reduced by reducing the stationary concentration of the aromatic ketone. It was shown that hydroxymethyl radicals are formed from methanol during the photolysis of TiO_2_ in the absence of additional acceptor compounds with formation of hydrogen and eventually formation of formaldehyde [46]. Both hydrogen and formaldehyde were also detected in our experiments by gas-phase analysis. Thus, higher amounts of hydroxymethyl radicals can be produced under lower concentration of the acceptor ketone and the probability of hydroxybenzyl radical dimerization (i.e., route II) is disfavoured under these conditions.

## Supporting Information

File 1 Experimental part.

## References

[1] Reckenthäler M, Griesbeck A G (2013). Adv Synth Catal.

[2] Xi Y, Yi H, Lei A (2013). Org Biomol Chem.

[3] Prier C K, Rankic D A, MacMillan D W C (2013). Chem Rev.

[4] Zou Y-Q, Chen J-R, Xiao W-J (2013). Angew Chem, Int Ed.

[5] König B (2013). Photoredox Catalysis.

[6] Hu J, Wang J, Nguyen T H, Zheng N (2013). Beilstein J Org Chem.

[7] Tucker J W, Stephenson C R J (2012). J Org Chem.

[8] Xuan J, Xiao W-J (2012). Angew Chem, Int Ed.

[9] Narayanam J M R, Jagan M R, Stephenson C R J (2011). Chem Soc Rev.

[10] Niwa T (2010). J Synth Org Chem, Jpn.

[11] Fujishima A, Rao T N, Tryk D A (2000). J Photochem Photobiol, C.

[12] Keck H, Schindler W, Knoch F, Kisch H (1997). Chem–Eur J.

[13] Hörner G, Johne P, Künneth R, Twardzik G, Roth H, Clark T, Kisch H (1999). Chem–Eur J.

[14] Hopfner M, Weiß H, Meissner D, Heinemann F W, Kisch H (2002). Photochem Photobiol Sci.

[15] Kisch H (2013). Angew Chem, Int Ed.

[16] Trost B M (2002). Acc Chem Res.

[17] Griesbeck A G, Reckenthäler M, Uhlig J (2010). Photochem Photobiol Sci.

[18] Griesbeck A G, Steinwascher J, Reckenthäler M, Uhlig J (2013). Res Chem Intermed.

[19] Sato T, Izumi G, Imamura T (1976). J Chem Soc, Perkin Trans 1.

[20] Sato T, Yoshiie S, Imamura T, Hasegawa K, Miyahara M, Yamamura S, Ito O (1977). Bull Chem Soc Jpn.

[21] Sato T, Yamaguchi S-i, Kaneko H (1979). Tetrahedron Lett.

[22] Sato T, Kaneko H, Yamaguchi S (1980). J Org Chem.

[23] Rossi B, Prosperini S, Pastori N, Clerici A, Punta C (2012). Molecules.

[24] Clerici A, Ghilardi A, Pastori N, Punta C, Porta O (2012). Org Lett.

[25] Kamigaito M, Sawamoto M, Higashimura T (1995). Macromolecules.

[26] Birse E F, McKenzie A, Murray A W (1988). J Chem Soc, Perkin Trans 1.

[27] Reetz M T, Westermann J, Steinbach R, Wenderoth B, Peter R, Ostarek R, Maus S (1985). Chem Ber.

[28] Wang A, Jiang H (2010). J Org Chem.

[29] Stocker J H, Kern D H (1968). J Org Chem.

[30] Lee J M, Park E J, Cho S H, Chang S (2008). J Am Chem Soc.

[31] Ortiz J, Guijarro A, Yus M (1999). Eur J Org Chem.

[32] Karthikeyan J, Jeganmohan M, Cheng C-H (2010). Chem–Eur J.

[33] Johnson T C, Totty W G, Wills M (2012). Org Lett.

[34] Uchiyama M, Matsumoto Y, Nakamura S, Ohwada T, Kobayashi N, Yamashita N, Matsumiya A, Sakamoto T (2004). J Am Chem Soc.

[35] Cleij M, Archelas A, Furstoss R (1999). J Org Chem.

[36] Chavan S P, Khatod H S (2012). Tetrahedron: Asymmetry.

[37] Li J, Wang C, Xue D, Wei Y, Xiao J (2013). Green Chem.

[38] DeGoey D A, Chen H-J, Flosi W J, Grampovnik D J, Yeung C M, Klein L L, Kempf D J (2002). J Org Chem.

[39] Itami K, Kamei T, Mitsudo K, Nokami T, Yoshida J-i (2001). J Org Chem.

[40] Ito S, Matsumoto M (1983). J Org Chem.

[41] Wang Z-M, Sharpless K B (1993). Synlett.

[42] Eliel E L, Bai X, Ohwa M (2000). J Chin Chem Soc.

[43] Sato T, Maeno H, Noro T, Fujisawa T (1988). Chem Lett.

[44] Bowker M (2011). Green Chem.

[45] Kohtani S, Nishioka S, Yoshioka E, Miyabe H (2014). Catal Commun.

[46] Micic O I, Zhang Y, Cromack K R, Trifunac A D, Thurnauer M C (1993). J Phys Chem.

