# Supplementary material for: Homogeneous and heterogeneous photoredox-catalyzed hydroxymethylation of ketones and keto esters: catalyst screening, chemoselectivity and dilution effects
Source: Beilstein J Org Chem. 2014 May 19;10:1143–50. doi: 10.3762/bjoc.10.114 (PMC4077468; doi:10.3762/bjoc.10.114)
Supplement: File 1 — Experimental part. [file Beilstein_J_Org_Chem-10-1143-s001.pdf]

Supporting Information

for

Homogeneous and heterogeneous photoredox-catalyzed hydroxymethylation of ketones and keto esters: catalyst screening, chemoselectivity and dilution effects

Axel G. Griesbeck* and Melissa Reckenthäler

Address: University of Cologne, Department of Chemistry, Organic Chemistry, Greinstr. 4, D-50939 Köln, Germany; Fax: +49(221)470 5057

Email: Axel G. Griesbeck - griesbeck@uni-koeln.de

* Corresponding author

Experimental part

General Methods

$TiCl(OiPr)_3$, $TiCl_2(OiPr)_2$ and $TiCl(OiPr)_3$ were prepared according to [1-3]. Methyl 6-oxoheptanoate was prepared following [4]. TiO_2 pigment was purchased from Kremer Pigmente, TiO_2 P25 was a gift from Degussa AG. All other chemicals were purchased from Acros Organics or Sigma-Aldrich and were used as received. ^{1}H NMR and ^{13}C NMR spectra were recorded on a Bruker AV 300 or Bruker DRX 500 spectrometer. HRMS were run on a Thermo Scientific LTQ Orbitrap XL mass spectrometer. All irradiations were carried out in pyrex or quartz vessels. All irradiation experiments except reactions done according to method b, were carried

out in a Luzchem LZ-C4 reactor (≈100 W) using coated and non-coated high-pressure mercury lamps (λ = 254, 300, 350 nm). In method b a Rayonet reactor (200 W) with coated high-pressure mercury lamps (λ = 300 nm) was used as light source.

Homogeneous catalyzed reactions

Argon gas was passed through a mixture of 0.75 mmol (1 equiv) ketone in 6 mL of methanol containing 0.5 equiv titanium(IV) catalyst of the type $TiCl_n(OiPr)_{4-n}$ (n = 0−4) for 15 min. The degassed solution was irradiated with UV light (254–300 nm) for 72 h at room temperature. After irradiation, the solution was poured into water and extracted four times with CH_2Cl_2. The organic phase was dried (Na_2SO_4) and the solvent was removed under reduced pressure. The residual was purified via column chromatography.

Heterogeneous catalyst reactions

Method a: Argon gas was passed through a mixture of 0.75 mmol (1 equiv) ketone in 6 mL methanol containing 1.4 wt % TiO_2 (0.5 mol % photoredox catalyst) for 15 min. The degassed solution was irradiated with UV light (350 nm) for 48 h at room temperature. After irradiation, the suspension was centrifuged to remove the nanoparticles. The supernatant solution was removed under reduced pressure and the residual was purified via column chromatography. In the case of acetophenone, the concentration of the substrate and product in supernatant was determined by a gas chromatograph (Hewlett-Packard HP-6890) equipped with flame ionization detector.

Method b: 3.72 mmol acetophenone dissolved in methanol (10 mL) was slowly added (441 µL/h) to a TiO_2 P25 (10 mg) in methanol (30 mL) suspension, while the reaction

solution was irradiated (300 nm) for 24 h at 15 °C. After irradiation, the suspension was centrifuged to remove the nanoparticles. The concentration of the substrate and product in supernatant was determined by a gas chromatograph (Hewlett-Packard HP-6890) equipped with flame ionization detector.

2-Phenylpropane-1,2-diol (**4**) [5]: ^{1}H-NMR ($CDCl_3$, 300 MHz): δ (ppm) 1.50 (s, 3H), 2.41 (brs, 1H, OH), 2.97 (brs, 1H, OH), 3.67 (dd, *J* = 47.3, 11.2 Hz, 2H), 7.26 (t, *J* = 7.1 Hz, 1H), 7.35 (t, *J* = 7.4 Hz, 2H), 7.43 (d, *J* = 7.4 Hz, 2H); ^{13}C-NMR ($CDCl_3$, 75 MHz): δ (ppm) 26.5 (q, C-1), 71.5 (t, C-7), 75.4 (s, C-2), 125.6 (d, C-5, C-5‘), 127.7 (d, C-6), 128.9 (d, C-4, C-4‘), 145.5 (s, C-3).

2,3-Diphenylbutane-2,3-diol (dl und meso-**5**) [6]: ^{1}H-NMR ($CDCl_3$, 300 MHz): δ (ppm) 1.51, (s, 6H), 1.58 (s, 6H), 7.18-7.26 (m, 20H); ^{13}C-NMR ($CDCl_3$, 75 MHz): δ (ppm) 24.7 (q), 24.9 (q), 126.6 (d), 126.8 (d), 126.9 (d), 127.0 (d), 127.1 (d), 143.1 (s), 143.5 (s).

1-Phenylethanol (**6**) [7]. ^{1}H-NMR ($CDCl_3$, 300 MHz): δ (ppm) 1.50 (d, *J* = 6.5 Hz, 3H), 1.86 (brs, 1H, OH) 4.91 (q, *J* = 6.4 Hz, 1H), 7.25-7.40 (m, 5H); ^{13}C-NMR ($CDCl_3$, 75 MHz): δ [ppm]: 25.7 (q), 71.0 (d), 125.9 (d), 128.1 (d), 129.1 (d).

1,1-Diphenylethane-1,2-diol (**7**) [8]. ^{1}H-NMR (d_4-MeOH, 300 MHz): δ (ppm) 4.11 (s, 2H), 7.21 (d, *J* = 7.2 Hz), 7.29 (t, *J* = 7.4 Hz), 7.44 (d, *J* = 7.3 Hz, 4H); ^{13}C-NMR (d_4-MeOH, 75 MHz): δ (ppm) 69.9 (t), 79.5 (s) 127.8 (d), 128.9 (d), 130.5 (d), 146.5 (s).

2-(2-Fluorophenyl)propane-1,2-diol (**10**). ^{1}H-NMR (300 MHz, $CDCl_3$): δ (ppm) 7.60 (t, *J* = 8.0 Hz, 1H), 7.25 (m, 1H), 7.14 (t, *J* = 7.5 Hz, 1H), 7.00 (m, 1H), 3.93 (d, J = 11.1 Hz, 1H), 3.69 (d, *J* = 11.2 Hz, 1H), 3.28 (s, 1H, OH), 2.51 (s, 1H, OH), 1.54 (s, 3H); ^{13}C-NMR (75 MHz, $CDCl_3$): δ (ppm) 161.2 (s), 157.9 (s), 131.7 (s), 131.6 (s), 129.3 (d), 129.2 (d), 128.2 (s), 128.2 (s), 124.4 (s), 124.3 (s), 116.3 (d), 115.9 (d), 74.2 (s), 74.2 (s), 69.4 (t), 69.3 (t), 24.7 (q), 24.7 (q)

2-(4-Nitrophenyl)propane-1,2-diol (**14**) [9]. ^{1}H-NMR (300 MHz, $CDCl_3$): δ (ppm) 8.19 (d, *J* = 8.8 Hz, 2H), 7.63 (d, *J* = 8.8 Hz, 2H), 3.80 (d, *J* = 11.0 Hz, 1H), 3.70 (d, *J* = 11.0, 1H), 2.41 (brs, 2H, OH), 1.55 (s, 3H); ^{13}C-NMR (75 MHz, $CDCl_3$): δ (ppm) 152.8 (s), 147.2 (s), 126.4 (d), 123.7 (d), 75.0 (s), 70.7 (t), 26.2 (q).

2-(*p*-Tolyl)propane-1,2-diol (**15**) [10]. ^{1}H-NMR (300 MHz, $CDCl_3$): δ (ppm) 7.32 (d, *J* = 8.2 Hz, 2H), 7.17 (d, *J* = 8.1 Hz, 2H), 3.73 (d, *J* = 11.1 Hz), 3.57 (d, *J* =11.0 Hz) 2.87 (brs, 2H, OH), 2.34 (s, 3H), 1.49 (s, 3H); ^{13}C-NMR (75 MHz, $CDCl_3$): δ (ppm) 142.2 (s), 136.9 (s), 129.9 (d), 125.2 (d), 74.9 (s), 71.2 (t), 26.2 (q), 21.1 (q).

2-Methylpentane-1,2-diol (**18**) [11]. ^{1}H-NMR ($CDCl_3$, 300 MHz): δ (ppm) 0.91 (t, *J* = 7.0 Hz, 3H), 1.13 (s, 3H), 1.29-1.48 (m, 4H), 2.79 (s, 2H, OH), 3.40 (q, *J* = 11.1 Hz, 2H, H-6); ^{13}C-NMR ($CDCl_3$, 75 MHz): δ (ppm) 14.8 (q), 17.1 (t), 23.2 (q), 41.1 (t), 69.7 (t), 73.3 (s).

1-(Hydroxymethyl)cyclohexanol (**19**) [12]. ^{1}H-NMR ($CDCl_3$, 300 MHz): δ (ppm) 1.25-1.66 (m, 10H), 1.92 (brs, 1H, OH), 2.10 (brs, 1H, OH), 3.45 (s, 2H); ^{13}C-NMR ($CDCl_3$, 75 MHz): δ (ppm) 21.6 (t), 25.6 (t), 34.1 (t), 69.9 (t), 71.5 (s).

1,1-Diphenylmethanol (**9**) [13]. ^{1}H-NMR ($CDCl_3$, 300 MHz): δ (ppm) 2.21 (brs, OH), 5.79 (s, 1H), 7.22-7.7.37 (m, 10H); ^{13}C-NMR ($CDCl_3$, 75 MHz): δ (ppm) 76.4 (d), 126.7 (d), 127.7 (d), 128.6 (d), 143.9 (s).

1-(2-Fluorophenyl)ethanol (**12**) [14]. ^{1}H-NMR (300 MHz, $CDCl_3$): δ (ppm) 7.51-7.45 (m, 1H), 7.27-7.20 (m, 1H), 7.17-7.11 (m, 1H), 7.04-6.98 (m,1H), 5.18 (q, *J* = 6.5 Hz, 1H), 2.26 (brs, 1H, OH), 1.50 (d, *J* = 6.5 Hz, 3H); ^{13}C-NMR (75 MHz, $CDCl_3$): δ (ppm) 161.5 (s), 158.3 (s), 132.9 (s), 132.7 (s), 128.9 (d), 128.8 (d), 126.8 (d), 126.8 (d), 124.5 (d), 124.5 (d), 115.6 (d), 115.3 (d), 64.7 (d), 64.6 (d), 24.2 (q).

1-(*p*-Tolyl)ethanol (**17**) [15]. ^{1}H-NMR (300 MHz, $CDCl_3$): δ (ppm) 7.26 (d, *J* = 7.9 Hz, 2H), 7.15 (d, *J* = 8 Hz, 2H), 4.85 (q, *J* = 6.4 Hz, 1H), 2.34 (s, 3H), 1.86 (brs, 1H, OH),

1.47 (d, J = 6.5 Hz, 3H): ^{13}C-NMR (75 MHz, $CDCl_3$): δ (ppm) 143.0 (s), 137.3 (s), 129.3 (d), 125.5 (d), 70.4 (d), 25.2 (q), 21.2 (q).

1,1,2,2-Tetraphenylethane-1,2-diol (**8**) [16]. ^{1}H-NMR ($CDCl_3$, 300 MHz): δ (ppm) 3.05 (s, 2H, OH), 7.18-7.19 (m, 12H), 7.30-7.33 (m, 8H).

2,3-Bis(2-fluorophenyl)butane-2,3-diol (**11**). ^{1}H-NMR (300 MHz, $CDCl_3$): δ (ppm) 7.49-7.43 (m, 2H), 7.31-7.05 (m, 8H), 7.04-6.80 (m, 6H), 3.25 (d, J = 6.2 Hz, 2H), 3.15 (d, J = 6.0 Hz, 2H), 1.77 (s, 6H), 1.64 (s, 6H); ^{13}C-NMR (75 MHz, $CDCl_3$): δ (ppm) 162.7 (s), 162.7 (s), 159.5 (s), 159.4 (s), 130.8 (t), 130.7 (t), 130.5 (t), 130.5 (t), 129.9 (t), 129.8 (t), 129.7 (t),124.1 (t), 124.1 (t), 123.7 (t), 123.7 (t), 116.9 (t), 116.6 (t), 116.5 (t), 116.3 (t), 80.2 (s), 80.2 (s), 80.1 (s), 80.0 (s), 24.9 (q), 24.9 (q), 24.5 (q), 24.4 (q); HR-MS: exact mass (ESI): m/z $[M+Na]^+$ cald. for $C_{16}H_{16}F_2O_2$: 301.1011 found: 301.1008

2,3-Bis(4-methoxyphenyl)butane-2,3-diol (dl and meso) (**13**) [17]. ^{1}H-NMR (300 MHz, $CDCl_3$): δ (ppm) 7.16-7.09 (m, 4H), 6.79-6.75 (m, 4H), 3.80 (s, 12H), [1.56] (s, 6H), 1.47 (s, 6H); ^{13}C-NMR (75 MHz, $CDCl_3$): δ (ppm) 158.5 (s), 158.4 (s), 136.0 (s), 135.7 (s), 128.5 (d), 128.1 (d), 112.6 (d), 112.4 (d), 78.7 (s), 78.5 (s), 55.2 (q).

2,3-Bis(4-methylphenyl)butane-2,3-diol (dl and meso) (**16**) [17]. ^{1}H-NMR (300 MHz, $CDCl_3$): δ (ppm) 7.19-7.06 (m, 16H), 2.36 (s, 6H), 2.35 (s, 6H), 1.56 (s, 6H), 1.48 (s, 6H); ^{13}C-NMR (75 MHz, $CDCl_3$): δ (ppm) 141.0 (s), 140.7 (s), 136.6 (s), 136.5 (s), 128.1 (d), 127.9 (d), 127.4 (d), 126.9 (d), 78.9 (s), 78.6 (s), 25.3 (q), 25.1 (q), 21.1 (q), 21.1 (q).

Methyl 2,3-dihydroxy-2-phenylpropanoate (**26**) [18]. ^{1}H-NMR (300 MHz, $CDCl_3$): δ (ppm) 7.60-7.55 (m, 2H), 7.41-7.29 (m, 3H), 4.26 (d, J = 11.5 Hz, 1H), 4.08 (brs, 1H, OH), 3.85 (s, 3H), 3.75 (d, J = 11.5 Hz, 1H), 2.53 (brs, 1H, OH); ^{13}C-NMR (75 MHz,

$CDCl_3$): δ (ppm) 174.3 (s), 138.2 (s), 128.6 (d), 128.5 (d), 125.5 (d), 79.9 (s), 68.4 (t), 53.7 (q).

Methyl 6,7-dihydroxy-6-methylheptanoate (**31**). ^{1}H-NMR (300 MHz, $CDCl_3$): δ (ppm) 3.62 (s, 3H), 3.37 (q, *J* = 11.0 Hz, 2H), 2.99 (s, 2H), 2.29 (t, *J* =7.4 Hz, 2H), 1.64-1.53 (m, 2H), 1.49-1.40 (m, 2H), 1.39-1.26 (m, 2H), 1.10 (s, 3H); ^{13}C-NMR (75 MHz, $CDCl_3$): δ (ppm) 174.4 (s), 72.9 (s), 69.6 (t), 51.6 (q), 38.2 (t), 33.9 (t), 25.5 (t), 23.3 (t), 23.1 (q); HR-MS: exact mass (ESI): m/z $[M+Na]^+$ cal. for $C_9H_{18}O_4$: 213.1097 found: 213.1096.

4-Hydroxy-4-phenyldihydrofuran-2(3*H*)-one (**28**) [19]. ^{1}H-NMR (300 MHz, d_4-MeOH): δ (ppm) 7.56 (d, *J* = 8.4 Hz, 2H), 7.42 (t, *J* = 7.4 Hz, 2H), 7.35 (d, *J* = 7.1 Hz, 1H), 4.48 (dd, *J* = 22.4, 10.0 Hz,), 2.99 (dd, *J* = 141.4, 17.1 Hz); ^{13}C (75 MHz, d_4-MeOH): δ (ppm) 177.7, 142.1, 129.3, 128.7,126.2, 80.7, 78.8, 44.2.

5-Hydroxy-5-methyltetrahydro-2*H*-pyran-2-one (**29**). ^{1}H-NMR (300 MHz, $CDCl_3$): δ (ppm) 3.62 (dd, *J* = 58.6, 12.1 Hz, 2H), 3.16 (brs, 1H, OH), 2.81-2.53 (m, 2H,), 2.44-2.33 (m, 1H), 2.01-1.86 (m, 1H), 1.38 (s, 3H); ^{13}C-NMR (75 MHz, $CDCl_3$): δ (ppm) 127.8 (s), 87.0 (s), 68.4 (t), 29.8 (t), 29.7 (t), 23.1 (q); HR-MS: exact mass (ESI): m/z $[M+Na]^+$ cald. for $C_6H_{10}O_3$: 153.0522 found: 153.0522.

6-(Hydroxymethyl)-6-methyltetrahydro-2*H*-pyran-2-one (**30**) [20]. ^{1}H-NMR (300 MHz, $CDCl_3$): δ (ppm) 3.56 (dd, *J* = 35.7, 11.9 Hz, 2H), 2.59-2.36 (m, 3H), 2.05-1.85 (m, 3H), 1.66-1.59 (m, 1H), 1.34 (s, 3H); ^{13}C-NMR (75 MHz, $CDCl_3$): δ (ppm) 171.6 (s), 84.6 (s), 69.4 (t), 29.7 (t), 28.7 (t), 23.5 (q), 16.9 (t); HR-MS: exact mass (ESI): m/z $[M+Na]^+$ cald. for $C_7H_{12}O_3$: 167.0679 found: 167.0678.

References

1. Kamigaito, M.; Sawamoto, M.; Higashimura, T. *Macromolecules* **1995,** *28,* 5671–5675. doi:10.1021/ma00120a037
2. Birse, E. F.; McKenzie, A.; Murray, A. W. *J. Chem. Soc., Perkin Trans. 1* **1988,** 1039–1046. doi:10.1039/p19880001039
3. Reetz, M. T.; Westermann, J.; Steinbach, R.; Wenderoth, B.; Peter, R.; Ostarek, R.; Maus, S. *Chem. Ber.* **1985,** *118,* 1421–1440. doi:10.1002/cber.19851180412
4. Ito, S.; Matsumoto, M. *J. Org. Chem.* **1983,** *48,* 1133–1135. doi:10.1021/jo00155a051
5. Wang, A.; Jiang, H. *J. Org. Chem.* **2010,** *75,* 2321–2326. doi:10.1021/jo100125q
6. Stocker, J. H.; Kern, D. H. *J. Org. Chem.* **1968,** *33,* 291–294. doi:10.1021/jo01265a057
7. Lee, J. M.; Park, E. J.; Cho, S. H.; Chang, S. *J. Am. Chem. Soc.* **2008,** *130,* 7824–7825. doi:10.1021/ja8031218
8. Ortiz, J.; Guijarro, A.; Yus, M. *Eur. J. Org. Chem.* **1999,** 3005–3012. doi:10.1002/(SICI)1099-0690(199911)1999:11<3005::AID-EJOC3005>3.0.CO;2-7
9. Cleij, M.; Archelas, A.; Furstoss, R. *J. Org. Chem.* **1999,** *64,* 5029–5035. doi:10.1021/jo982101+
10. Chavan, S. P.; Khatod, H. S. *Tetrahedron: Asymmetry* **2012,** *23,* 1410–1415. doi:10.1016/j.tetasy.2012.09.008
11. DeGoey, D. A.; Chen, H.-J.; Flosi, W. J.; Grampovnik, D. J.; Yeung, C. M.; Klein, L. L.; Kempf, D. J. *J. Org. Chem.* **2002,** *67,* 5445–5453. doi:10.1021/jo0162890
12. Itami, K.; Kamei, T.; Mitsudo, K.; Nokami, T.; Yoshida, J. *J. Org. Chem.* **2001,** *66,* 3970–3976. doi:10.1021/jo015528g
13. Karthikeyan, J.; Jeganmohan, M.; Cheng, C.-H. *Chem.–Eur. J.* **2010,** *16,* 8989–8992. doi:10.1002/chem.201001160
14. Johnson, T. C.; Totty, W. G.; Wills, M. *Org. Lett.* **2012,** *14,* 5230–5233. doi:10.1021/ol302354z
15. Li, J.; Wang, C.; Xue, D.; Wei, Y.; Xiao, J. *Green Chem.* **2013,** *15,* 2685–2689. doi:10.1039/c3gc41133h
16. Clerici, A.; Ghilardi, A.; Pastori, N.; Punta, C.; Porta, O. *Org. Lett.* **2012,** *10,* 5063–5066. doi:10.1021/ol802244n
17. Uchiyama, M.; Matsumoto, Y.; Nakamura, S.; Ohwada, T.; Kobayashi, N.; Yamashita, N.; Matsumiya, A.; Sakamoto, T. *J. Am. Chem. Soc.* **2004,** *126,* 8755–8759. doi:10.1021/ja039674a
18. Wang, Z. M.; Sharpless, K. B. *Synlett* **1993,** 603–604. doi:10.1055/s-1993-22547
19. Eliel, E. L.; Bai, X.; Ohwa, M. *J. Chin. Chem. Soc.* **2000,** *47,* 63–70.
20. Sato, T.; Maeno, H.; Noro, T.; Fujisawa, T. *Chem. Lett.* **1988,** *10,* 1739–1742. doi:10.1246/cl.1988.1739
